# Serum zonulin, brain-derived neurotrophic factor, and oxidative stress levels in individuals using paper (A4) impregnated with synthetic cannabinoids

**DOI:** 10.3389/fpsyt.2025.1693270

**Published:** 2025-10-23

**Authors:** Sibel Kulaksızoğlu, Ali Erdoğan, Oğuzhan Tosun, Zeynep Yaren Görgülü, Esra Acar, Yavuz Kireç, Sercan Karabulut, Burak Kulaksızoğlu, Buket Cinemre, Özmen Metin

**Affiliations:** ^1^ Department of Medical Biochemistry, Antalya Education and Research Hospital, Antalya, Türkiye; ^2^ Department of Psychiatry, Akdeniz University Faculty of Medicine, Antalya, Türkiye

**Keywords:** synthetic cannabinoid, A4, zonulin, BDNF, oxidative stress

## Abstract

**Introduction:**

A4 is paper impregnated with synthetic cannabinoids, used as a novel psychoactive substance. The aim of this study was to investigate serum zonulin, brain-derived neurotrophic factor (BDNF), total oxidant status (TOS), total antioxidant status (TAS) and oxidative stress index (OSI) in patients predominantly using synthetic cannabinoid-impregnated papers.

**Methods:**

Thirty patients with A4 use and 30 healthy volunteers (HC) were included. Peripheral blood was collected for zonulin, BDNF, TOS, TAS and OSI levels after 8–12 hours of fasting. Substance Craving Scale (SCS) was administered to the patient group.

**Results:**

Patients using A4 had lower BDNF levels (p=0.001) and higher TOS and OSI levels (p<0.001, p<0.001) compared to HC. Zonulin and TAS levels of both groups were similar (p>0.005 for both). No correlation was found between the SCS scores and zonulin, BDNF, TAS, TOS and OSI values (p>0.05 for all). When ROC analysis was performed, the cut-off value for BDNF in the patient group was ≤2.51 pg/mL, sensitivity was 66.7%, specificity was 63.3%, PPV was 76.0%, NPV was 76.0%, and area under the ROC curve was 0.744. The cut-off value for TOS was ≥14.51 μmol/L, sensitivity 70.0%, specificity 66.7%, PPV 77.7%, NPV 79.5%, area under the ROC curve 0.766. The cut-off value for OSI was ≥0.69, sensitivity 76.7%, specificity 63.3%, PPV 76.0%, NPV 81.3%, area under the ROC curve 0.773.

**Discussion:**

The findings suggest that A4 use may impair neuroplasticity and disrupt oxidative stress balance. BDNF, TOS, and OSI appear to be promising biomarker candidates for identifying individuals with A4 use.

## 1 Introduction

Substance use disorder (SUD) is a significant public health problem. According to the 2024 United Nations World Drug Report, the number of people using substances increased to 292 million in 2022. This means a 20% increase in the last 10 years. Cannabis remains the most widely used drug worldwide, with 228 million users (1). Cannabis (marijuana) primarily contains natural cannabinoids such as Δ9-tetrahydrocannabinol (Δ9-THC) and cannabidiol (CBD). In contrast, synthetic cannabinoids are novel psychoactive substances that also act on the endocannabinoid system but usually as full agonists at cannabinoid receptors, leading to more potent and potentially more harmful effects than natural cannabis. The metabolism of synthetic cannabinoids differs from that of classical cannabis. Synthetic cannabinoids do not undergo chemical conversion to classical cannabis metabolites such as 11-OH-THC or THC-COOH; instead, they are biotransformed via CYP450 enzymes into structurally distinct metabolites. Synthetic cannabinoids are generally metabolized by CYP450 enzymes, particularly CYP3A4, CYP2C9, and CYP2E1, but their metabolites are highly diverse. Unlike THC, they can produce hundreds of structural variants. Most synthetic metabolites are pharmacologically inactive; however, some have been reported to possess toxic potential. These metabolic products are not detected by standard toxicology screening and require advanced analytical methods such as LC–MS/MS or HRMS. Moreover, certain metabolites may contribute to oxidative stress and mitochondrial toxicity, leading to more harmful biochemical effects compared to THC (2–4). Increasing intoxications and deaths associated with synthetic cannabinoids have emerged as an important public health problem in recent years (5–7). However, to date, no epidemiological data are available that specifically report mortality rates associated with synthetic cannabinoids. In recent years, A4 paper impregnated with synthetic cannabinoids has been reported as a form of synthetic cannabinoid use in Turkey. Paper soaked or sprayed with solvents containing synthetic cannabinoids is used by organized crime groups as a method to transport synthetic cannabinoids into prisons (8). The use of A4, a synthetic cannabinoid-impregnated paper, is increasing rapidly in our country (9).

Various biochemical parameters in SUD have been studied in the literature. One of these is oxidative parameters. In a meta-analysis evaluating stimulants, alcohol, nicotine, opioids, cannabis, inhalants and polysubstance use, higher levels of oxidant markers and lower levels of antioxidant markers were found in SUD than in healthy controls (10). In a study comparing individuals with cannabis use disorder and healthy controls, increases in total oxidant status (TOS) and oxidative stress index (OSI) values were found, but no difference was found in total antioxidant status (TAS) levels. It has been found that cannabis use disrupts the oxidative balance and creates oxidative stress (11).

Another biochemical parameter that is emphasized in psychiatric diseases is brain-derived neurotrophic factor (BDNF). It has been studied as a potential biomarker in various psychiatric disorders, including SUD. It has been reported that patients who use substances have lower serum BDNF levels than healthy controls (12, 13). However, there are also studies reporting that BDNF levels are higher in SUD (14).

In addition to neurotrophic factors such as BDNF, alterations in systemic inflammation and intestinal barrier function have also been implicated in the pathophysiology of SUD. Intestinal barrier damage caused by SUD allows harmful substances and bacteria to cross into the circulatory system, leading to systemic inflammatory responses and immune imbalances (15). A recently popular molecule related to the intestinal barrier is zonulin, where increased levels indicate enhanced intestinal permeability (16). However, there is currently no literature evaluating zonulin levels in SUD patients.

The aim of this study was to compare serum levels of zonulin, BDNF, TAS, TOS, and OSI between patients with A4 use (patients with SUD predominantly consuming synthetic cannabinoid-impregnated papers) and healthy controls (HC).

## 2 Methods

### 2.1 Sample and procedure

This study was designed as a single-center cross-sectional case-control study. A total of 30 patients who applied to Akdeniz University Alcohol and Substance Addiction Research and Treatment Center (AMBAUM) with SUD according to DSM-5 diagnostic criteria (SUD; predominantly using synthetic cannabinoid-impregnated papers, A4) and 30 healthy controls (HC) matched for age and gender were included in the study. Sample size calculation was conducted using the G*Power 3.1 software (17). Based on data from a reference study (10), a power analysis was performed to compare the means of two independent groups using a t-test. Assuming a large effect size (Cohen’s d = 0.80), an alpha level of 0.05, and a statistical power of 0.80 (1–β), the minimum required sample size was determined to be 26 participants per group (a total of 52 participants).

#### 2.1.1 Inclusion criteria for the patient group

Meeting the diagnostic criteria for SUD according to DSM-5 predominantly involving synthetic cannabinoid-impregnated papers (A4), using only A4 at the last application, no other substance use in the last 6 months being between the ages of 18-65, voluntarily accepting to participate in the study after being informed.

#### 2.1.2 Exclusion criteria for the patient group

Psychiatric disorder comorbidity (e.g., psychotic disorder, bipolar disorder, schizoaffective disorder, etc.), presence of physical disease (e.g., liver disease, kidney disease, cardiovascular disease, cancer, etc.), history of neurological disease, presence of intellectual disability, use of antiinflammatory drug and antioxidant drug in the last two weeks before the study.

#### 2.1.3 Inclusion criteria for the HC group

Healthy controls were required to be between 18 and 65 years of age, have no history of psychiatric or physical illness, and report no previous use of alcohol or other substances.

#### 2.1.4 Exclusion criteria for the HC group

In the healthy control group, exclusion criteria included a history of neurological disease, presence of intellectual disability, and use of anti-inflammatory or antioxidant medications within two weeks preceding the study.

All participants completed the Sociodemographic data form and the Substance Craving Scale (SCS) (18). Ethical approval was obtained from the Clinical Research Ethics Committee of the University of Health Sciences Antalya Training and Research Hospital (Decision No: 5/23, dated 25.04.2024). Written informed consent forms were obtained from all participants. Our study was conducted in accordance with the Helsinki Declaration.

### 2.2 Blood sample collection and analysis

Approximately 10 mL of venous blood was collected from the antecubital vein of each participant between 08:00 and 10:00 AM following an overnight fast. Samples were collected in biochemical tubes containing gel. Serum was obtained by centrifuging at 3000 rpm for 10 minutes in a Nuve NF800 centrifuge device. Samples were stored at -80 °C until the day of the study. After all samples were collected, serum BDNF and zonulin levels were measured simultaneously by enzyme-linked immunosorbent assay (ELISA) method, and TAS and TOS levels were measured by spectrophotometric method. Biochemical analyses were performed in the Antalya Education and Research Hospital Medical Biochemistry Laboratory. Serum BDNF levels were measured using a BDNF ELISA Kit (BT LAB, Cat. No. E1302Hu, China), and zonulin levels were measured using a Zonulin ELISA Kit (BT LAB, Cat. No. E3704Hu, China). TAS levels were determined with commercially available colorimetric kits (Rel Assay Diagnostic, Gaziantep, Turkey). This automated method is based on the bleaching of the characteristic color of the stable ABTS [2,2′-azino-bis (3-ethylbenzothiazoline-6-sulfonic acid)] radical cation by antioxidants. The test sensitivity was <3%, and results were expressed as mmol Trolox equivalent/L. TOS levels were also determined using commercially available colorimetric kits (Rel Assay Diagnostic, Gaziantep, Turkey) (19). The repeatability of all assays was CV <10%. Samples and reagents were mixed and kinetic readings were made on a spectrophotometer after 10 minutes. Sample analysis was performed using the Oxidant Status kit (Relassay, Türkiye) (20) on a fully automated AU 5800 biochemistry autoanalyzer.

### 2.3 Statistical analysis

Continuous variables are given as mean ± standard deviation, median (Q1-Q3) and Mean Rank, categorical data are given as numbers and percentages. Normality analyses of continuous variables were performed using Kolmogorov-Smirnov Goodness of Fit Test. Mann Whitney U Test was used in the analysis of data that did not conform to normal distribution. Chi-square Test (Fisher’s Exact Test when necessary) was used in the comparison of categorical data. Linear relationship between scales was performed using Spearman Correlation Analysis. In the patient group, Area Under the Curve (AUC) values ​​and cut-off values ​​were examined with Receiver Operating Characteristics (ROC) curve analysis to determine whether BDNF, TOS and OSI values ​​were a variable that could be used in diagnosis. Analyses were performed using IBM SPSS (Statistical Package for Social Sciences) version 23.0 (IBM Corporation, Armonk, NY, USA). Statistical significance level was accepted as p<0.05.

## 3 Results

The mean age was 29.63 ± 7.14 years in patient group and 27.00 ± 7.08 years in the HC group (p=0.138). All participants were male. The clinical characteristics of the patient group are presented in **Table 1**.

**Table 1:** Sociodemographic and clinical characteristics of the patient group.

		n	%
Forensic case history	Yes	17	56.7
	No	13	43.3
Application to psychiatric clinics other than addiction centers	Yes	15	50.0
	No	15	50.0
A4 usage frequency	Several times a month	2	6.7
	Several times a week	3	10.0
	Every day	25	83.3
History of methamphetamine use	Yes	7	23.3
	No	23	76.7
History of heroin use	Yes	4	13.3
	No	26	86.7
History of cocain use	Yes	7	23.3
	No	23	76.7
History of cannabis use	Yes	14	46.7
	No	16	53.3
History of alcohol use	Yes	14	46.7
	No	16	53.3
History of pregabalin abuse	Yes	10	33.3
	No	20	66.7
Number of criminal cases (median)		1.00	

The comparison of the patient group and the HC group in terms of zonulin, BDNF, TAS, TOS and OSI levels is summarized in **Table 2**.

**Table 2:** Comparison of patient and healthy control groups in terms of biochemical parameters.

	A4 group (n=30)	Healthy control (n=30)	P
Zonulin*			
Median (Q1-Q3) Mean Rank	17.56 (14.61-29.43) 33.65	18.01 (11.42-20.07) 27.35	0.162
Brain derived neurotrophic factor (BDNF)*			
Median (Q1-Q3) Mean Rank	2.12 (1.79-2.88) 23.17	2.63 (2.35-4.11) 37.83	**0.001**
Total oxidant status (TOS)*			
Median (Q1-Q3) Mean Rank	27.65 (9.43-47.97) 38.47	6.10 (1.48-17.17) 22.53	**<0.001**
Total antioxidant status (TAS)*			
Median (Q1-Q3) Mean Rank	1.22 (1.11-1.35) 30.20	1.20 (1.06-1.45) 30.80	0.894
Oxidative stress index (OSİ)			
Median (Q1-Q3) Mean Rank	2.03 (0.68-3.90) 38.70	0.49 (0.10-1.54) 22.30	**<0.001**

*BDNF: pg/mL, Zonulin: ng/mL, TAS: mmol /L, TOS: μmol/L. The bold values indicate statistically significant results (p < 0.05).

The ROC analysis conducted in the patient group to determine whether BDNF, TOS and OSI values are a variable that can be used in diagnosis is summarized in **Table 3**.

**Table 3:** Cut-off values and ROC analysis results for BDNF, TOS and OSI values in the A4 group.

	Diagnostic testing					ROC curve		*P*
	Cut-off value	Sensitivity	Specificiy	PPV*	NPV*	AUC*	%95 CI*	
BDNF^&^	≤ **2.51**	66.7	63.3	76.0	76.0	**0.744**	0.616-0.873	**0.001**
TOS^&^	**≥14.51**	70.0	66.7	77.7	79.5	**0.766**	0.646-0.885	**<0.001**
OSI	**≥0.69**	76.7	63.3	76.0	81.3	**0.773**	0.656-0.890	**<0.001**

*PPV, Positive predictive value; NPV, Negative predictive value; AUC, Area under the curve; CI, Confidence interval.

^&^BDNF: pg/mL, TOS: μmol/L.

The bold values indicate statistically significant results (p < 0.05).

The zonulin, BDNF, TAS, TOS and OSI values of patients with (n=19) and without (n=11) previous history of cannabis, heroin, cocaine, methamphetamine and pregabalin use were found to be similar (p>0.05 for all). No correlation was found between SCS scores and zonulin, BDNF, TAS, TOS and OSI values (p>0.05 for all). OSI values were significantly higher in patients with a criminal history (median=3.57) than in those without (median=1.70) (p=0.043).

## 4 Discussion

**Figure 1:**
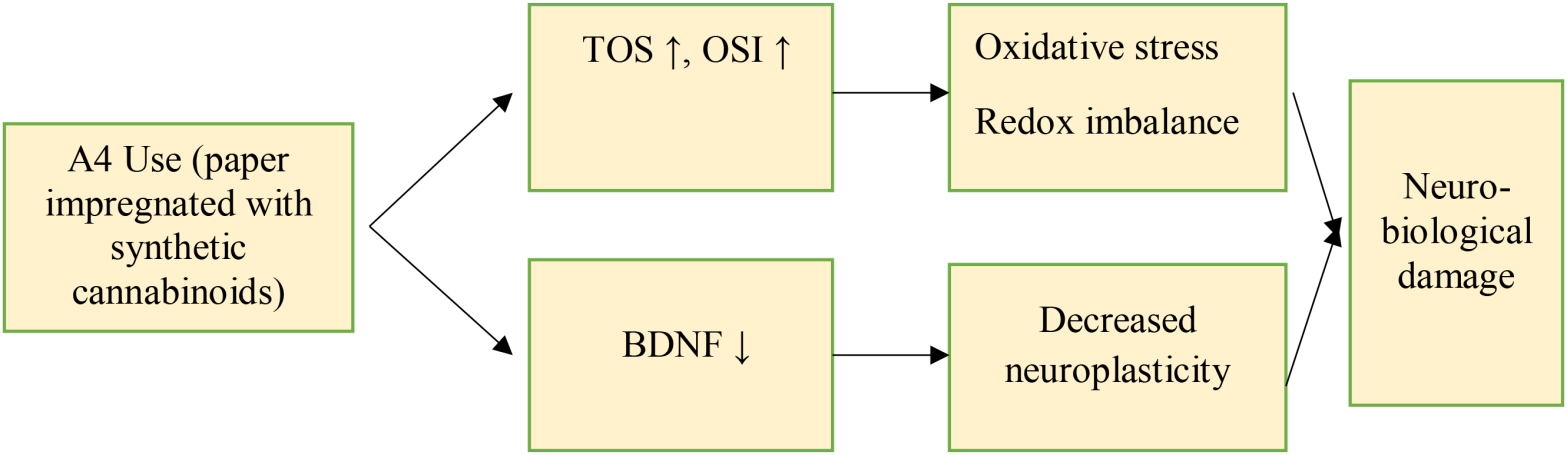
Proposed pathophysiological mechanisms underlying A4 use in this study.

In our study, the patient group (SUD with predominant A4 use) showed significantly reduced BDNF levels and elevated TOS and OSI levels compared to HCs; in contrast, zonulin and TAS levels did not differ between groups. There was no correlation between craving score and biochemical parameters. Based on the ROC analysis, the cut-off values were found to be ≤2.51 pg/mL for BDNF, ≥14.51 μmol/L for TOS and ≥0.69 for OSI (**Figure 1**).

Neurotrophins are a family of proteins important for many functions in the nervous system and are involved in cell growth and differentiation. Among neurotrophins, BDNF has been one of the most well-established. It is an extracellular signaling molecule promoting neurons development, plasticity and survival (21). BDNF has been examined in several psychiatric disorders and has been proposed as a putative biomarker in several disorders, including SUD (12). In a meta-analysis of 40 studies including 2238 patients with SUD and 2574 HCs, serum BDNF levels were found to be significantly reduced in active users compared to HCs. However, this divergence appeared to diminish during remission. Subgroup analysis revealed that BDNF levels were particularly lower in patients using alcohol and crack/cocaine, suggesting that BDNF may be a putative biomarker of interest in these populations (13). A further meta-analysis, consisting of 11 studies, revealed that changed blood BDNF levels were not significantly associated with cannabis use, suggesting a requirement of additional studies in order to elucidate the interactions among cannabis use and the neurotrophic factors (22). A study on 45 cannabis-dependent patients and 45 HCs found more elevated BDNF levels in patients with cannabis use disorder. These authors hypothesized that this increase may reflect a compensatory mechanism due to a loss of neuronal plasticity in addiction (14). In our research, patients had lower serum BDNF levels. This finding may suggest an impaired neuroplasticity and neuronal damage in central nervous system in A4 users. The finding of reduced BDNF in patients vs. healthy controls indicates that BDNF may be a useful biomarker in this population. Our findings suggest that a BDNF cut-off value of ≤ 2.51 pg/mL may be a reliable indicator of A4 use. However, these results should be supported by studies with larger samples.

Another important result of our research was the increased TOS, and OSI levels, which are important markers of oxidative stress, in patients with A4 use. In contrast, TAS were similar between patients and HCs. Recent studies have suggested that central and peripheral toxicities of SUD might be partially associated with a imbalance in the production of reactive oxygen species and the antioxidant defense systems. A meta-analysis reported higher oxidant markers and lower antioxidant markers in SUD patients than in HCs (6). In a study comparing 34 patients with cannabis use disorder and 34 HCs, it was found that patients with cannabis use disorder had higher levels of TOS, OSI, interleukin 1β, IL-6, IL-8, and tumor necrosis factor α compared to HCs. TAS, IL-12, and interferon γ levels were similar in both groups (11). The balance between oxidants and antioxidants is crucial for brain aging. Free radicals in the body cause oxidative damage to cellular and genetic material, leading to tissue and organ dysfunction, and ultimately resulting in death (11, 23, 24). All substance use can lead to oxidative stress and may contribute to SUD-related toxicity in the brain and peripheral nervous system. Additionally, the higher levels of oxidant markers compared to antioxidants at different stages of treatment in SUD patients can lead to cell dysfunction and death through the oxidation of DNA, proteins, or phospholipids. This can have significant clinical implications for early morbidity and mortality (25, 26). Our findings also indicate that there is an oxidative imbalance in patients with A4 use that can lead to cell damage and multiple morbidities, including accelerated aging. Indeed, while TAS levels are similar to HCs, TOS and OSI levels are much higher than those of HCs. This indicates that patients using synthetic cannabinoids (A4) are exposed to intense oxidative stress. Although oxidant markers were elevated in A4 users, antioxidant levels remained unchanged. This finding may suggest that increased oxidant production exceeded the buffering capacity of the antioxidant defense system, or that chronic substance use may weaken adaptive antioxidant responses. In addition, inflammatory markers such as CRP, IL-1β, IL-6, IL-8, and TNF-α, and antioxidant enzymes including glutathione peroxidase, catalase, and superoxide dismutase, play an important role in oxidative stress and immune regulation (27–29). Although these markers were not assessed in our study, they should be considered in future research to provide a more comprehensive understanding of the biochemical alterations associated with A4 use.

In our study, A4 users exhibited significantly reduced BDNF levels, together with elevated TOS and OSI, while TAS remained unchanged. This pattern may indicate that chronic A4 exposure impairs neuroplasticity and neuronal survival, reflected by decreased BDNF, whereas increased oxidant production exceeds the buffering capacity of the antioxidant defense system, leading to oxidative imbalance without a measurable rise in total antioxidant status. Unlike Δ9-THC, which is a partial agonist, most synthetic cannabinoids are full agonists of cannabinoid receptors. This results in greater efficacy, with more intense psychoactive effects and more severe side effects. Furthermore, synthetic cannabinoids blends lack cannabidiol, a compound present in cannabis that modulates cannabinoid receptor activity and helps counterbalance the psychoactive effects of Δ9-THC. The chemical structure of synthetic cannabinoids in A4 differs markedly from classical cannabis, generating numerous structurally diverse metabolites through CYP450 metabolism. Several of these metabolites have been associated with mitochondrial toxicity and enhanced reactive oxygen species production (7, 30). Taken together, these findings suggest a potential mechanism in which A4 use leads to excessive oxidative stress and impaired antioxidant responses, ultimately contributing to neurotoxicity and reduced BDNF levels. This mechanistic pathway may help explain the severe clinical consequences observed with A4 use and highlights the need for further translational studies.

In our study, the cut-off values were determined as ≥14.51 μmol/L for TOS and ≥0.69 for OSI. Particularly, BDNF along with TOS and OSI could be potential biomarkers for detecting the use of synthetic cannabinoids, which are extremely difficult to identify and pose significant issues in forensic psychiatry.

In our study, no difference was found in zonulin levels. Zonulin is a significant factor in intestinal permeability. It is a protein that increases intestinal permeability in the small intestine and contributes to the innate immunity of the gut. It is produced by the human small intestinal epithelium under the influence of environmental stimuli (31, 32). Substance use causes damage to the intestinal barrier. The intestinal barrier damage caused by SUD allows harmful substances and bacteria to pass through the intestinal barrier and enter the circulatory system, leading to systemic inflammatory responses and immune imbalances (15). However, there is no literature available that evaluates zonulin levels in SUD patients. In our study, zonulin levels were found to be similar to those of healthy controls. This may suggest that synthetic cannabinoids alone may not be sufficient to alter intestinal permeability or that intestinal permeability may also be influenced by other variables. Ultimately, intestinal permeability can be affected by many factors (diet, medications, lifestyle, infections) (33).

### 4.1 Strengths and limitations

Our study has several limitations. These include the relatively small sample size, the cross-sectional design, and the fact that all participants were male. Although we included only patients who had used A4 during the past six months, many had a history of prior psychoactive substance use. Since other substances are also known to influence the biomarkers (11) examined in our study, it is difficult to fully disentangle the potential effects of prior substance use, and this has been acknowledged as a limitation.

The strengths of our study are that it is the first in the literature to evaluate BDNF, oxidative stress, and zonulin levels in A4 users, and that it also included a control group.

## 5 Conclusion

It can be said that there is a decrease in neuroplasticity and an increase in oxidative stress in patients, while there is no change in intestinal permeability. We suggest that BDNF, TOS and OSI levels can be used as biomarkers to detect synthetic cannabinoid use, especially at certain cut-off points. Considering the insufficient literature on this subject, prospective, large-sample and multicenter studies are needed where each substance is evaluated separately. Future studies should incorporate inflammatory biomarkers to provide a more comprehensive understanding of the pathophysiological mechanisms underlying A4 use. We also recommend that future studies include female users to investigate whether hormonal levels contribute to the redox effects of A4.

## Data Availability

The raw data supporting the conclusions of this article will be made available by the authors, without undue reservation.
